# Arthroscopic Treatment of Femoral Avulsion Fracture of the Posterior Cruciate Ligament in Association with Meniscus Tear

**DOI:** 10.1111/os.12636

**Published:** 2020-03-10

**Authors:** Xin Zhao, Shi‐da Kuang, Chao Su, Wen‐feng Xiao, Guang‐hua Lei, Shu‐guang Gao

**Affiliations:** ^1^ Department of Orthopaedics Xiangya Hospital, Central South University Changsha China; ^2^ National Clinical Research Center of Geriatric Disorders Xiangya Hospital, Central South University Changsha China; ^3^ Center for Clinical Technology and Research of Joint Surgery of Hunan Province Changsha China

**Keywords:** Femoral avulsion fracture, Knee injuries, Meniscus tear, Posterior cruciate ligament

## Abstract

**Background:**

Femoral avulsion fracture of the posterior cruciate ligament (PCL) in association with meniscus tear is extremely rare in children, and similar cases are not available in the existing literature.

**Case presentation:**

In this paper, we present a case of a 9‐year‐old boy treated by an arthroscopic repair technique using two transfemoral tunnels sparing the epiphyseal plate for 8‐shaped suture fixation of femoral avulsion fracture of the PCL and using the Fastfix repair system for a meniscus tear. At 3‐month postoperative, the boy showed satisfactory recovery with a full range of motion of the right knee and normal muscular strength, and no complications were observed after operation. The patient had been followed up for 24 months and his final assessment demonstrated stable painless knee with excellent functional outcome. In view of this, we suggest that the “figure‐of‐eight” suture technique using two transfemoral tunnels sparing the epiphyseal plate can be considered a new choice for the treatment of the PCL femoral attachment avulsion, especially in skeletally immature patients. In addition, six similar cases were found in a comprehensive literature review targeting femoral avulsion fracture of the PCL. According to the relevant findings and cases studies, we proposed a new classification named “Xiangya” which might facilitate future clinical decision making.

## Introduction

In general, posterior cruciate ligament (PCL) injuries can be classified into several patterns according to the anatomic lesion, including mid‐substance failure, tibial avulsion, femoral peel‐off, and femoral avulsion. Injuries to the PCL usually involve ligament substance or tibial insertion and, less commonly, avulsion from the femoral insertion^1^. While several reports demonstrated femoral avulsion in adults, very limited cases in children have been published. This may be partly attributed to the fact that osteochondral avulsions of the PCL can be easily misdiagnosed on plain radiographs in children^2^. In recent years, arthroscopic treatments of avulsion fractures from the tibial insertion of the PCL have been reported^2^, ^3^, ^4^, ^5^, but publications on the femoral avulsion of the PCL are still rare^1^, ^6^, ^7^, ^8^, ^9^, ^10^, ^11^, ^12^, and a “gold standard” of treatment is not yet available. This paper presented an extremely rare case of a PCL avulsion from the femoral insertion in association with a meniscus tear as well as its arthroscopic management in a child. To support our research, a comprehensive literature review was conducted, which retrieved six similar cases. There were no relevant reports on the classification of PCL femoral avulsion fracture, and, therefore, we proposed a new classification of PCL femoral avulsion fracture named “Xiangya”.

Specifically, this study was performed with the following purposes: (i) to provide a new technique for the treatment of PCL femoral attachment avulsion especially in skeletally immature patients; and (ii) to explore a new classification of PCL femoral avulsion fracture to facilitate clinicians in making more appropriate surgery decisions for patients with PCL femoral attachment avulsion.

Our targeted case corresponds to a boy with PCL avulsion from the femoral insertion in association with a meniscus tear who received treatment in our hospital. Written informed consent had been obtained from the patient prior to the research alongside all the related clinical reports with detailed clinical diagnosis and treatment data. PubMed and Science Direct databases were searched using the following key words: “femoral avulsion fracture” and “posterior cruciate ligament”. Due to the low‐incidence, large‐sample summative studies, and with randomized controlled trials still lacking, all the relevant publications that exist as case reports. Therefore, the included literature is mainly based on detailed records of the patients' specific conditions, treatment processes, and follow‐up articles. For this reason, the patients' basic characteristics and treatment plans were analyzed.

## Case Presentation

**Figure 1:**
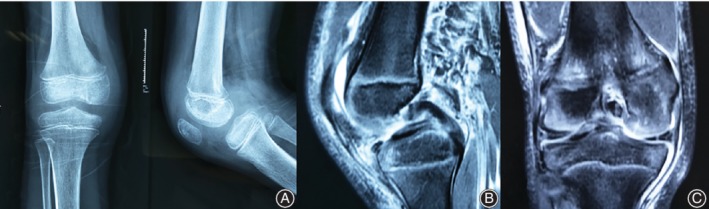
Preoperative X‐ray film showing a thin avulsed fragment of the posterior cruciate ligament arising from the femoral attachment (A). Preoperative MRI of the right knee showing an avulsion of the PCL at the femoral insertion (B) and a meniscus tear in the body of medial meniscus (C).

A 9‐year‐old boy injured his right knee in a fall while riding a motorcycle. He complained of pain and swelling, and was admitted to our hospital 7 days after the incident. Detailed physical examination was difficult to carry out due to the pain. Plain radiography did not reveal femoral avulsion fracture of the PCL. X‐ray images of the right knee showed a displaced femoral avulsion fracture of the PCL (Fig. 1A). On the magnetic resonance image (MRI), a bony avulsion of the PCL was observed at the femoral insertion (Fig. 1B) and a meniscus tear was found in the body of medial meniscus (Fig. 1C). An arthroscopy examination of the knee joint was performed 14 days after the injury.

*Anesthesia and Position*. General anesthesia was executed in the operation with the patient being placed in a dorsal position. His right knee showed a positive result of posterior drawer test under general anesthesia, and >1 cm posterior sagging was observed in comparison with the left knee.

**Figure 2:**
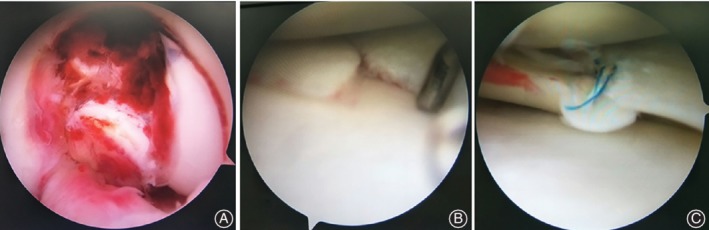
Intraoperative arthroscopic views from anterior portals of the displaced femoral avulsion fracture of the PCL (A), a meniscal tear in the body of medial meniscus (B) and the subsequent meniscus repair (C).

A thigh tourniquet was typically used to control bleeding and improve visualization. Routine anterolateral and anteromedial portals were created for intraarticular surgery. The arthroscopic view was obtained from an anterolateral portal. Hematoma and large blood clots in the joint were carefully removed to expose the femoral avulsion fracture (Fig. 2A) of the PCL. Partial synovectomy was performed to ensure adequate visualization and facilitate the reduction of the avulsed bone fragment into its bed.

Meniscus tear was identified and probed with a small metal hook (Fig. 2B). There was no damage to the articular cartilage or substance of the ACL. The fracture fragment, which was approximately 1 cm × 1 cm in size (Fig. 2A), restricted full flexion of the knee, and was attached with all the fibers of the PCL in the notch. This loose fragment was separated from the medial femoral condyle. Corresponding defect in the medial femoral condyle was found (Fig. 2A).

**Figure 3:**
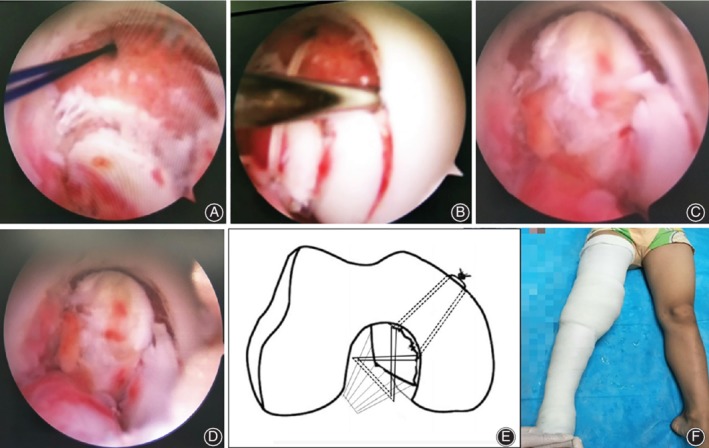
Sketches of the surgical technique, the arthroscopy photographs and the long leg plaster cast for postoperative external fixation. (A) Intraoperative arthroscopic views from anterior portals of the displaced femoral avulsion fracture of the PCL; a hole was drilled with a 2‐mm Kirschner wire (K‐wire) toward the medial epicondyle and a double‐stranded looped PDS was inserted into the joint through the epidural cannula. (B) The white suture loop was pulled through the PDS loop out of the anteromedial portal with a line grasping forceps; another hole was drilled with a 2‐mm Kirschner wire (K‐wire) toward the medial epicondyle and a second white suture loop was pulled through the PDS loop. The avulsion fracture was treated with arthroscopic‐assisted reduction (C) and was tightly fixed (D). The “figure‐of‐eight” of this kind of suture technique (E). The long leg plaster cast for postoperative external fixation (F).

The radial tear in the body of medial meniscus was repaired with the Fastfix meniscus repair device before reduction of the femoral avulsion fracture (Fig. 2C). Then, the fragment was probed and elevated, allowing the surgeon to inspect any comminution that might influence reduction or fixation. Two tunnels were drilled by using a 2‐mm K‐wire toward the medial epicondyle (one from the upper inner area of the fracture edge and another from the outside area of the fracture edge) while sparing the epiphyseal plate, and a guide pin connected with a double stranded looped PDS (Fig. 3A) was inserted into the anterolateral portal. The guide pin was then removed. Thereafter, two strands of No. 2 Ultrabraid sutures (Smith & Nephew, Andover, MA) were placed into the joint and the sutures were crossed from the back of the PCL to the front. The white suture loop was pulled through the PDS loop out of the anteromedial portal with a line grasping forceps. A 2‐cm‐long medial incision was then made and the white sutures were pulled into the incision by the double‐stranded, looped PDS (Fig. 3B,C). Finally, the avulsed PCL was reduced in 90° of knee flexion and the sutures were tightened and tied onto the medial condylar bone surface (Fig. 3D). We regard this kind of suture technique as the “figure‐of‐eight” suture, as it visually resembles the shape of the numeral “8” (Fig. 3D).

After the surgery, the patient was required to wear a plaster cast for 2 weeks to heal the fracture (Fig. 3F), and functional exercises were enabled after removing the plaster cast. Early recovery of muscle strength was encouraged for the first 6 weeks, and full weight bearing was allowed at 6 weeks postoperatively. The follow‐up time points were specified as 1, 3, 12, and 24 months after operation. The main contents of follow‐up include symptom evaluation, stability, joint range of motion, and complications.

The operation lasted for about 90 min with a total bleeding loss of about 15 ml. It was found that the osteochondral fracture was stable in full range of motion (ROM) after being fixed, the articular surface had recovered its smoothness under arthroscopy, and the PCL reattachment held tension on testing (Fig. 3E). A posterior draw‐sign was attempted, which showed a negative result. No major perioperative complications were reported during the study period.

**Figure 4:**
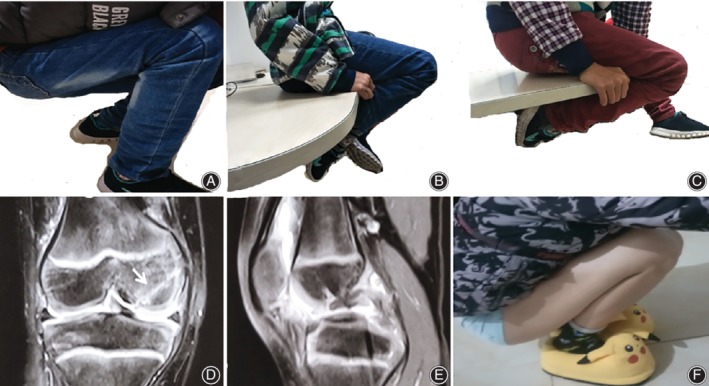
The brace was removed after 2 weeks, and full ROM was allowed at 1–month (A), 2–month (B), 3–month (C) and 24–month (F) postoperatively. The MR images obtained at 3‐month postoperatively indicate that the bone tunnel (white arrow) and the avulsion fracture are in correct positions with smooth articular surface (D, E).

At 1 month postoperative, the pain and claudication symptoms had completely disappeared. Flexion of the knee was approximately 110° at 1 month (Fig. 4A), 125° at 2 months (Fig. 4B), 140° at 3 months (Fig. 4C), and recovered to normal at 24 months after surgery(Fig. 4F). At 3‐month follow‐up, the patient had returned to normal life activities without pain or restrictions, and the Lysholm score had been improved from 31 points preoperatively to 84 points. Meanwhile, physical examination showed a negative sign of posterior drawer test, and MRI demonstrated consolidation of osteochondral fragment (Fig. 4D,E). Neither bone tunnels nor sutures passed through the epiphysis. At 12‐month and 24‐month follow‐up, the patient feedback was that he was satisfied with the operation outcome.

Forty‐six similar cases were found from the literature search and six cases involving femoral attachment avulsion fractures in children were retained (an overview was given in Table 1).

**Table 1:** Comparison of PCL femoral attachment avulsion fractures in children

Study	Year	Age (years)	Sex	Physical examination	Surgery	Fragment(cm)	Follow‐up(months)	Result
Mayer and Micheli^7^	1979	11	Boy	Tenderness swelling, effusion, ADS(+),PDS(+)	Sutures passed through drill holes across the medial condyle	_	12	Stable painless knee with an excellent functional outcome
Hesse *et al*. 1	2006	9	Boy	Effusion,PDS(+),	A transosseous femoralfixation using non‐absorbable stitches	1 × 1	12	Intact peripheral perfusion and sensation. The gait was physiological
Mirzatolooei^13^	2010	8	Boy	Flexion deformity swelling ecchymosis on the popliteal fossa	No surgery and he received physical therapy	1 × 0.5	12	The patient had a normal hip and near‐full knee flexion. He had no rotary instability and was active in sport.
Itokazu *et al*.^8^	1990	12	Boy	A 20° loss of extension and a slight posterior laxity	The osteochondral fragment was extirpated arthroscopically	1 × 1.5	_	Two days later the boy was able to walk freely and did not complain of pain, subjective instability, restriction of motion, or limitation of activity
Sanders *et al*.^14^	1980	6	Boy	Effusion tenderness, PDS(+)	By means of two Steinmann pins, perforated to accept a suture and passed through the defect in the medial femoral condyle	1 × 1.5	24	Returned to all of his usual activities but pain after vigorous activity. Had a 5‐degree loss of extension and a 15‐degree loss of flexion and slight posterior laxity
Sanders^14^	1980	8	Boy	Effusion tenderness ADS(+),PDS(+)	The avulsed posterior cruciate ligament was reattached as it was in patient 5	1 × 1	–	The patient was in the rehabilitation phase of treatment at the time of writing

ADS, anterior drawer sign; PDS, posterior drawer sign.

## Discussion

The PCL is approximately twice as strong as the anterior cruciate ligament (ACL) and represents the strongest ligament of the knee^15^. It plays an important role in stabilizing the knee joint. Since the majority of avulsed fragments were found in the medial femoral condyle, we assumed that the chondro‐osseous junction of the femoral attachment was the weakest point of the PCL in a child. However, this presumption needs to be validated by more confirmatory data.

Up to the present, practical experience in the treatment of femoral avulsion fractures of the PCL for children is limited due to the low incidence of such injuries. Although some similar cases were reported in the literature, a “gold standard” is not yet available^7^, ^8^, ^15^, ^16^, ^17^. To sum up, an osteochondral fragment may be treated by the traditional open surgery or arthroscopy, which involves procedures using screws, cerclage, non‐absorbable stitches, or K‐wires. In addition, autologous chondrocyte implantation, or the use of an osteochondral autograft or allograft, had also been reported. It is noteworthy that the case presented in this paper involves a new technique that has never been reported before.

After a comprehensive search, six cases in adolescents were found in the literature (Table 1), including five cases treated operatively (but only one patient received arthroscopic treatment) and one treated non‐operatively because of delayed diagnosis. Earlier cases have reported conservative treatment in the PCL femoral original avulsion patients but showed different outcomes^14^, ^15^. Gillespie and Crawford treated two patients non‐operatively and obtained poor functional results, while Mirzatolooei reported a case with satisfactory recovery after physical therapy^13^, ^14^. In our opinion, it is better to opt for surgery treatment rather than risking the early knee laxity associated with poor function or late arthritis^1^.

There was no consensus about whether to use a cast or brace after surgery. According to Table 1, Patient 2 wore a long leg cast and a knee brace for 6 weeks in total, Patient 5 was immobilized for 8 weeks in a cast, and Patient 6 wore a cast for a total of seven weeks. For the remaining patients, the related publications did not disclose whether a cast or brace was applied postoperatively. In contrast, our patient was protected with only a plaster cast for 2 weeks after surgery, and the cast was removed together with the incision sutures. At the 24‐month follow‐up, we found that the patient had been able to take part in sporting activities without crutches and his gait had gradually returned to its normal physiological actions.

The case presented in our study featured somewhat unusual characteristics. First, the avulsion occurred at the femoral origin of the PCL. Second, the fragment could be fixed regardless of its shape or size by using two transfemoral tunnels sparing the epiphyseal plate for 8‐shaped suture fixation. This new technique overcame the disadvantage of screws which could only fix large fracture fragments. Third, our “figure‐of‐eight” suture technique can be regarded as an improvement on previous techniques by providing perfect stability to the knee to allow earlier functional exercises. Fourth, growth arrest or angular deformity related to physeal injury must be taken into consideration during the operation. Our technique avoided distal femoral physis when placing the medial femoral condylar drill holes, and the fragment was fixed without disrupting future growth potential.

The advantages of this technique include the ability to diagnose and treat concomitant injuries, anatomic reduction of the fragment and stabilization in a limited surgical time, as well as early rehabilitation with full weight bearing. Last but not least, there is no need for device removal. Although controversy still exists, we believe that the physeal‐sparing technique can address the concerns about growth disturbance to a large extent. To the best of our knowledge, this is the first study that investigates the arthroscopic treatment of displaced femoral avulsion fracture of the PCL by using two transfemoral tunnels sparing the epiphyseal plate for 8‐shaped suture fixation. Of course, we have also noticed some deficiencies in the present study. It had been only 24 months since the operation, and follow‐up is still in progress.

**Figure 5:**
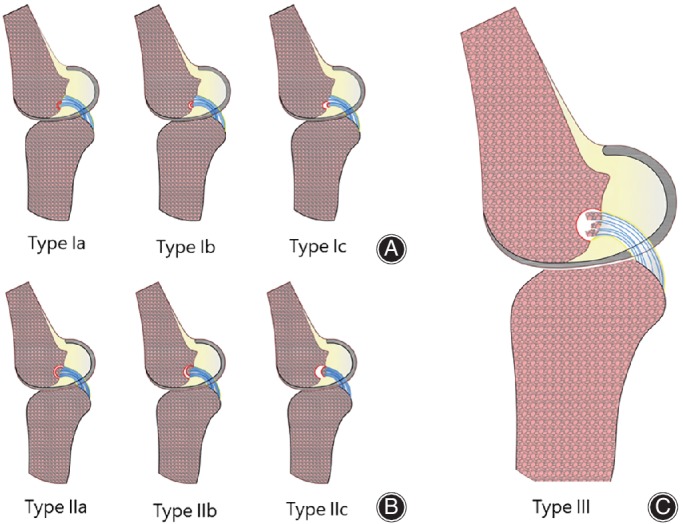
Xiangya classification of the PCL femoral avulsion fracture. Type I: the single avulsion fracture fragment area < half of the PCL femoral origin; Type Ia: the fragment has no displacement; Type Ib: the fragment has only a minimal displacement; Type Ic: injuries demonstrate complete separation of the fragment from the femur; Type II: the single avulsion fracture fragment area > half of the PCL femoral origin while the subtype classification (IIa，IIb，IIc) is the same as type I; Type III: injuries include a rotational component or comminution of the fragment.

At present, no relevant reports have been found on the classification of PCL femoral avulsion fracture. Therefore, we proposed a new classification of PCL femoral avulsion fracture named “Xiangya”, which depended mainly on the size and displacement degree of the fracture fragment. We hoped this classification would provide some reference for future clinical decision making and treatment of PCL femoral attachment avulsion fractures (Fig. 5). Specifically, Type Ia, Ib, IIa, and IIb fractures can be treated conservatively, while for type Ic, IIc, and III fractures, there are indications of surgical treatment.

The present study involves several limitations. Firstly, we only focused on one case, and there was the lack of a randomized controlled study, so more cases are needed to further validate the effectiveness of this new technique. Secondly, our case's follow‐up period was relatively short. It is necessary to execute a longer term follow‐up to evaluate growth disturbances. Nevertheless, the follow‐up for our case is 24 months, which is deemed sufficiently robust to report on the clinical outcomes. Thirdly, the practicability of this new “Xiangya” classification needs to be tested by further clinical practice.

In conclusion, our “figure‐of‐eight” suture technique using two transfemoral tunnels sparing the epiphyseal plate can be a new choice of treatment for the PCL femoral attachment avulsion, especially in skeletally immature patients.
